# Customized Closed‐Tray Impression Post for Unknown Implant System: Case Report

**DOI:** 10.1155/crid/3673906

**Published:** 2026-02-23

**Authors:** Turki S. Alkhallagi

**Affiliations:** ^1^ Oral and Maxillofacial Prosthodontics Department, Faculty of Dentistry, King Abdulaziz University, Jeddah, Saudi Arabia, kau.edu.sa

**Keywords:** closed-tray impression transfer copings, customized implant laboratory analog, customized impression post, definitive implant prosthesis, implant-supported crown, unknown dental implant system

## Abstract

Managing implants with unknown systems presents a significant clinical challenge when obtaining an accurate master impression. This case report describes a simple technique for fabricating a customized closed‐tray implant impression post and laboratory analog when the implant system cannot be identified. The use of these customized components enabled accurate transfer of the implant position and facilitated the fabrication of a definitive prosthesis. This case report highlights a practical solution for clinicians facing similar cases involving unknown implant systems.

## 1. Introduction

Dental implants have become a predictable and widely accepted treatment modality for replacing missing teeth, with numerous implant systems available in the market [1]. However, complications arise when the dental implant system is unknown, especially in cases where the implant was placed by another dentist, incomplete documentation, or when implant systems have been discontinued. The increasing number of implant manufacturers is another factor that increases the prevalence of this clinical challenge.

Accurate identification of the implant system is essential during prosthetic rehabilitation, as selection of compatible impression and restorative components depends on precise implant‐abutment matching. When the implant system is unknown, conventional workflow is difficult or impossible to perform. This complicates impression‐making and increases the risk of prosthetic inaccuracies.

Accurate impression‐making is critical for the long‐term success of implant prostheses [2]. Conventional closed‐tray and open‐tray impression techniques are widely used and require system‐specific impression posts and laboratory analogs [2, 3]. In cases involving unknown implant systems, the use of standard impression components may be impossible.

Several alternative solutions have been proposed to address this clinical challenge. Digital workflows involving cone beam computed tomography (CBCT), reverse engineering, and implant recognition software can assist in identifying the implant system or addressing this clinical challenge [4–8]. However, these methods rely on advanced digital tools and specialized software which may not be available in every clinical setting.

This case report presents a practical technique for fabricating a customized closed‐tray impression post and laboratory analog for an unknown implant system. By adapting available materials and following conventional workflow, an accurate master impression was achieved, leading to a successful and precise definitive prosthesis.

## 2. Case Presentation

A 68‐year‐old female patient presented with mobility of a zirconia cement‐retained implant‐supported crown replacing the maxillary right second premolar. Her medical history was noncontributory, with no known allergies or systemic conditions contraindicating dental treatment. The implant had been placed 9 years earlier; however, the patient had no records identifying the implant system, and the previous dental clinic was no longer in operation.

**Figure 1:**
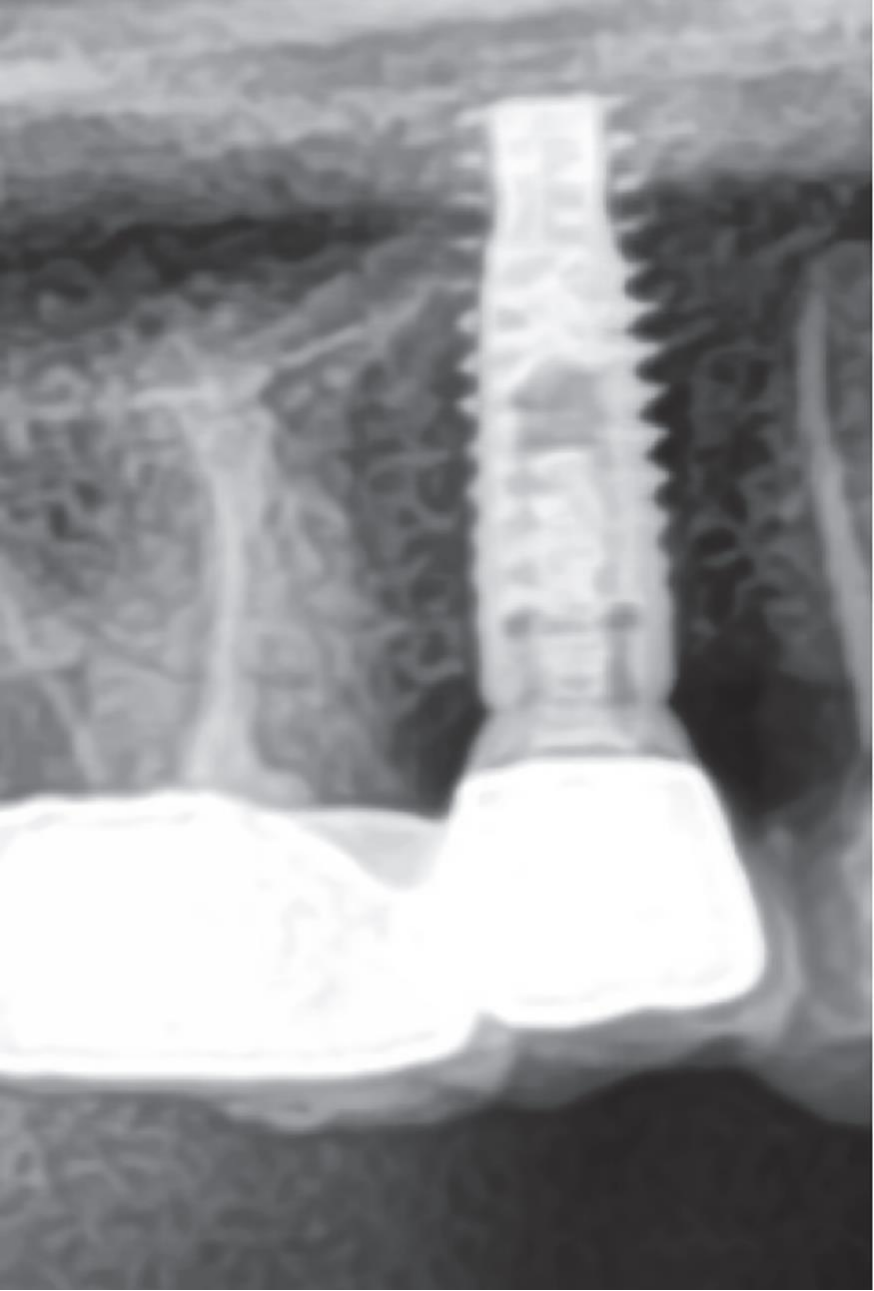
Preoperative radiographic image showing the implant with adequate peri‐implant bone support and no evidence of marginal bone loss prior to crown removal.

Clinical examination revealed healthy peri‐implant soft tissues with no signs of infection or inflammation. The implant was clinically stable, and the only mechanical complication noted was crown mobility. A radiographic image demonstrated adequate bone around the implant and absence of marginal bone loss (Figure 1). Occlusal analysis identified excessive lateral contacts on the implant crown, which were considered a contributing factor to prosthetic screw loosening.

The implant system could not be identified using radiographs or other standard methods through its shape, connection, or dimensions [4]. Furthermore, artificial intelligence‐based recognition tools were used to identify this implant, but the identification process was not successful [4, 6]. Because the implant system remained unknown, standard impression post and laboratory analog were not possible.

The treatment plan involved the removal of the loose cement‐retained crown and fabrication of a new screw‐retained crown. This treatment approach facilitates retrievability and reduces the risk of future screw fracture, which is the most common technical complication in single implant‐supported crowns [9]. To achieve this plan, the previous abutment was utilized in conjunction with self‐cured acrylic resin to fabricate the customized closed‐tray impression post and laboratory analog. The customized components were designed to replicate the geometry and function of standard closed‐tray impression post and laboratory analog, allowing accurate transfer of the implant position during impression‐making.

A definitive polyvinyl siloxane (PVS) impression was obtained using the customized components, followed by fabrication and delivery of a definitive screw‐retained implant crown. At the 1‐year follow‐up visit, clinical and radiographic evaluations demonstrated stable peri‐implant soft tissue, maintained marginal bone level, and excellent prosthesis function. The integration of these findings has been strengthened to emphasize long‐term success.

## 3. Procedure

**Figure 3:**
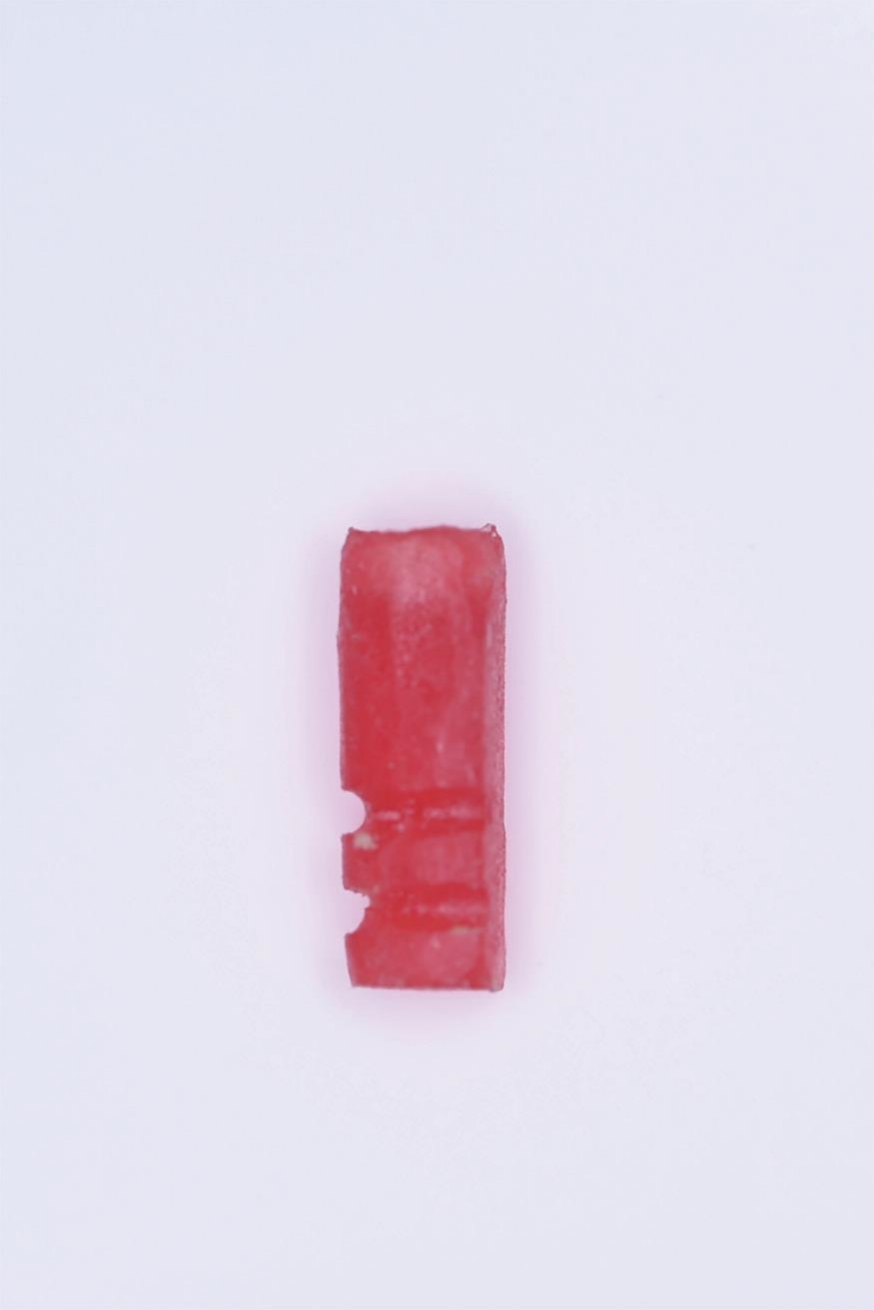
Customized laboratory analog designed with two horizontal grooves to enhance retention within the definitive cast and prevent displacement.

**Figure 5:**
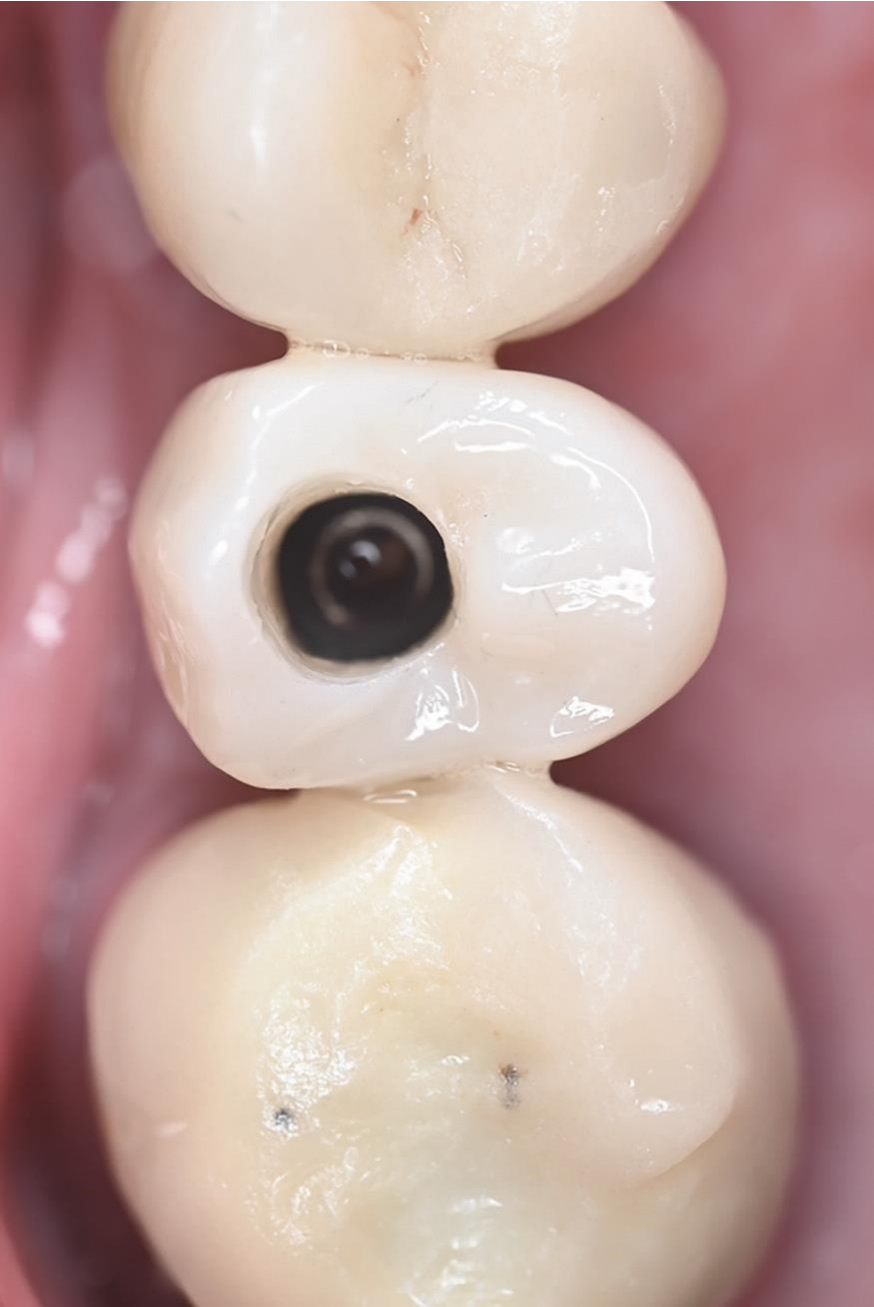
A definitive screw‐retained implant‐supported zirconia crown (occlusal view) delivered and torqued, demonstrating proper seating and alignment.

A. Remove the loose cement‐retained implant‐supported crown by sectioning it without damaging the existing implant abutment. Then, unscrew the abutment using the universal screwdriver kit. Examine the existing abutment and central screw. Both were in good condition, with sufficient axial wall height in the existing abutment.
B. Fabricate a customized closed‐tray impression post using self‐cured acrylic resin (GC Dental Pattern Resin, GC, Luzern, Switzerland) over this existing abutment following the manufacturer’s recommendation (Figure 2A). Adjust the customized closed‐tray impression post to include three vertical grooves and an inclined bevel, which prevents rotation within the impression.
C. Fabricate a customized laboratory analog using self‐cured acrylic resin (GC Dental Pattern Resin, GC, Luzern, Switzerland) in contact with the existing abutment connection following the manufacturer’s recommendation (Figure 3). Adjust the customized laboratory analog to include two horizontal grooves, which improve its retention in the master cast. Then, check the abutment stability on the customized laboratory analog.
D. Place the customized closed‐tray impression post and check it intraorally and radiographically (Figure 2B).
E. Take a standard closed‐tray impression using PVS material (Zhermack SpA, Badia Polesine, Italy) (Figure 4). After complete setting, remove the impression carefully from the patient’s mouth, followed by the customized closed‐tray impression post.
F. Insert the customized laboratory analog into the abutment. Insert the customized closed‐tray impression post with the customized laboratory analog into the impression (Figure 4). Then, pour the master impression with type IV dental stone to create a definitive cast.
G. Remove the customized closed‐tray impression post from the abutment. Scan the master cast with the abutment in place using Ceramill Map 400+ scanner (AmannGirrbach, GmbH for Ceramill Map 400 Scanner, Koblach, Austria).
H. Design the definitive prosthesis as a screw‐retained implant‐supported zirconia crown over the abutment using Exocad software (Exocad, Darmstadt, Germany).
I. Mill the definitive crown (Prime Zirconia, Ivoclar Vivadent, Liechtenstein, Switzerland) using CORiTEC 250i Loader Pro system (imes‐icore, Eiterfeld, Germany). Then, cement the definitive prosthesis to the abutment using resin cement (3M RelyX Unicem).
J. Check and try‐in the definitive prosthesis intraorally and radiographically for fit, occlusion, and esthetics (Figure 5). Adjust the occlusion of the definitive prosthesis as necessary.
K. Provide the patient with postoperative care instructions.

Figure 2Customized closed‐tray impression post designed with three vertical grooves and an inclined bevel to enhance rotational stability and ensure accurate repositioning after impression‐making. (A) Shows customized closed‐tray impression post design. (B) Shows customized closed‐tray impression post screwed onto implant (occlusal view), demonstrating proper intraoral fit.

**Figure:**
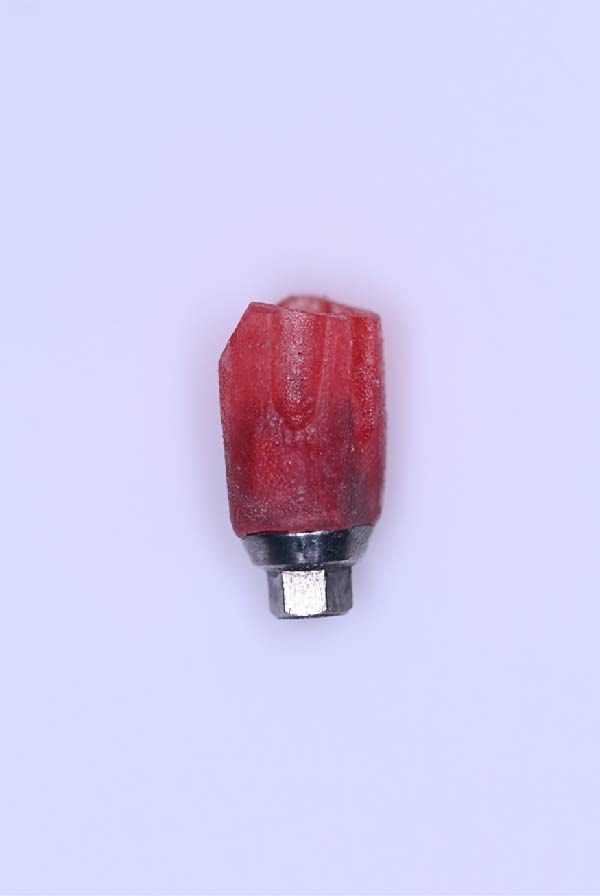
(A)

**Figure:**
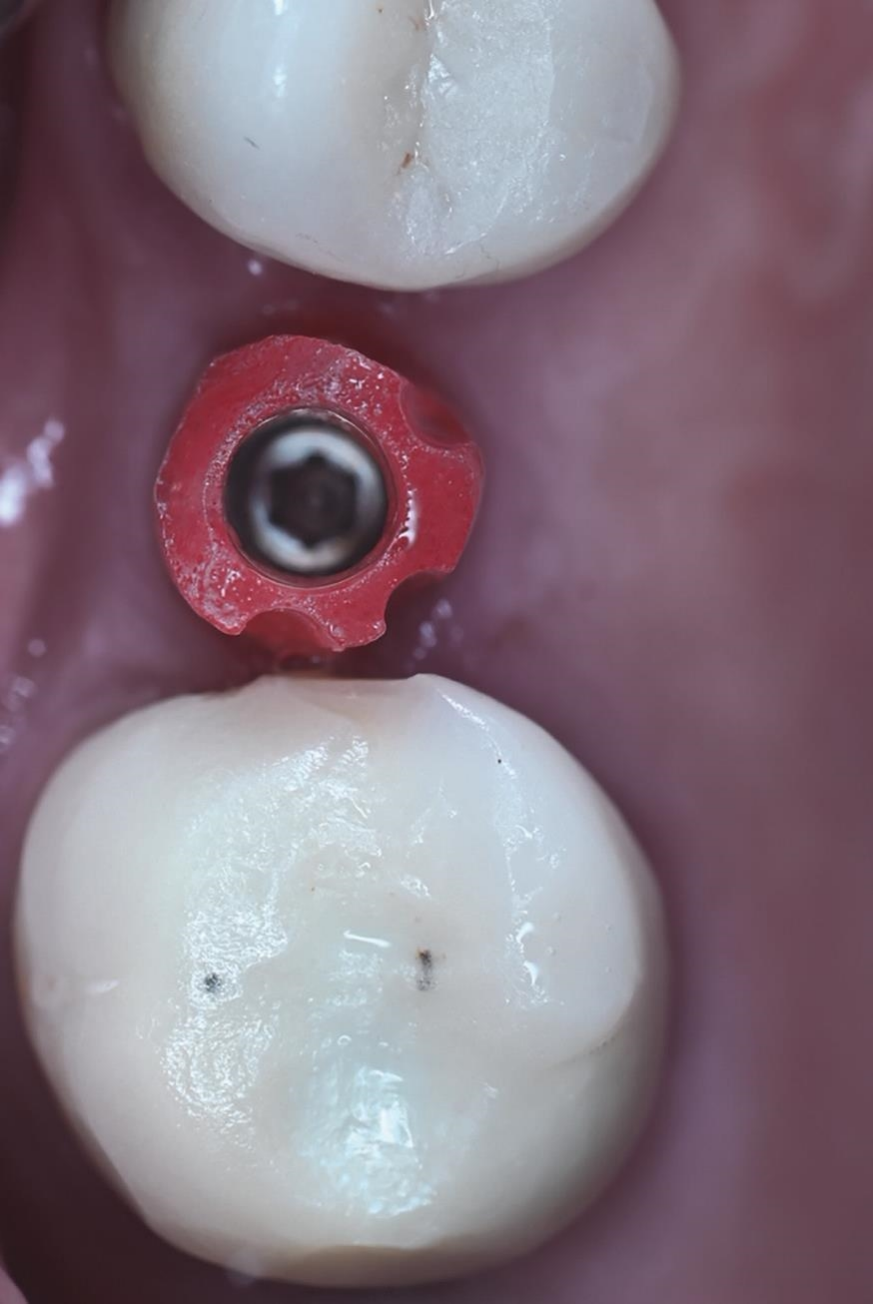
(B)

Figure 4Master impression incorporating three vertical protrusions to allow accurate transfer of implant orientation. (A) Shows closed‐tray master impression without the customized closed‐tray impression post. (B) Shows customized closed‐tray impression post and customized laboratory analog positioned within the master impression.

**Figure:**
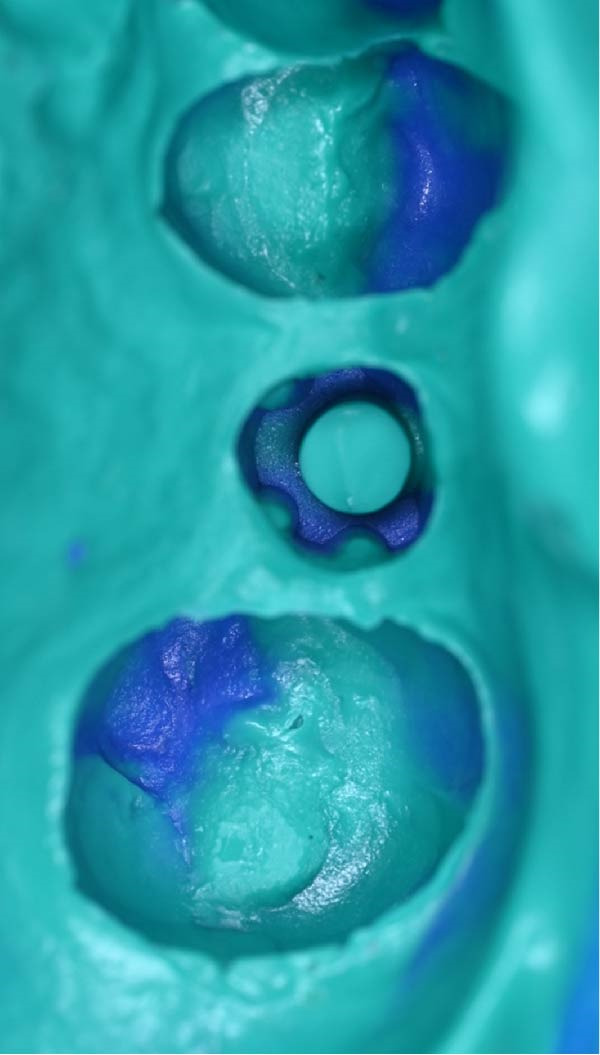
(A)

**Figure:**
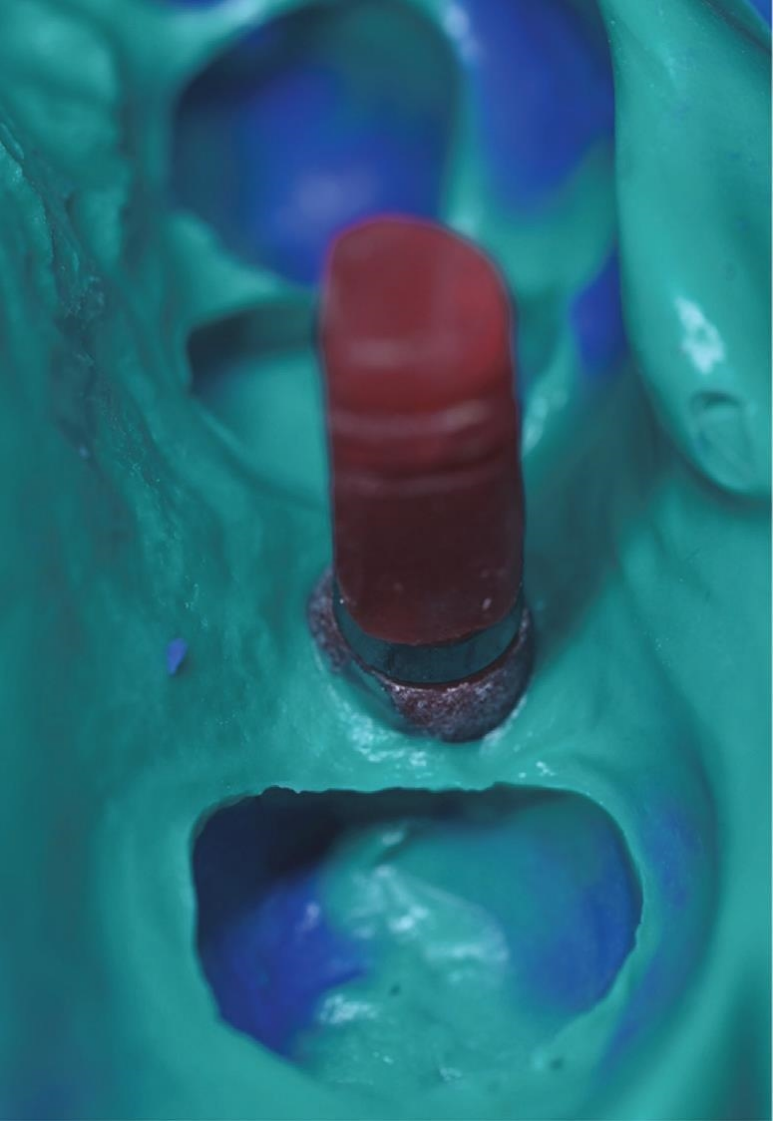
(B)

## 4. Follow‐Up and Case Outcomes

**Figure 6:**
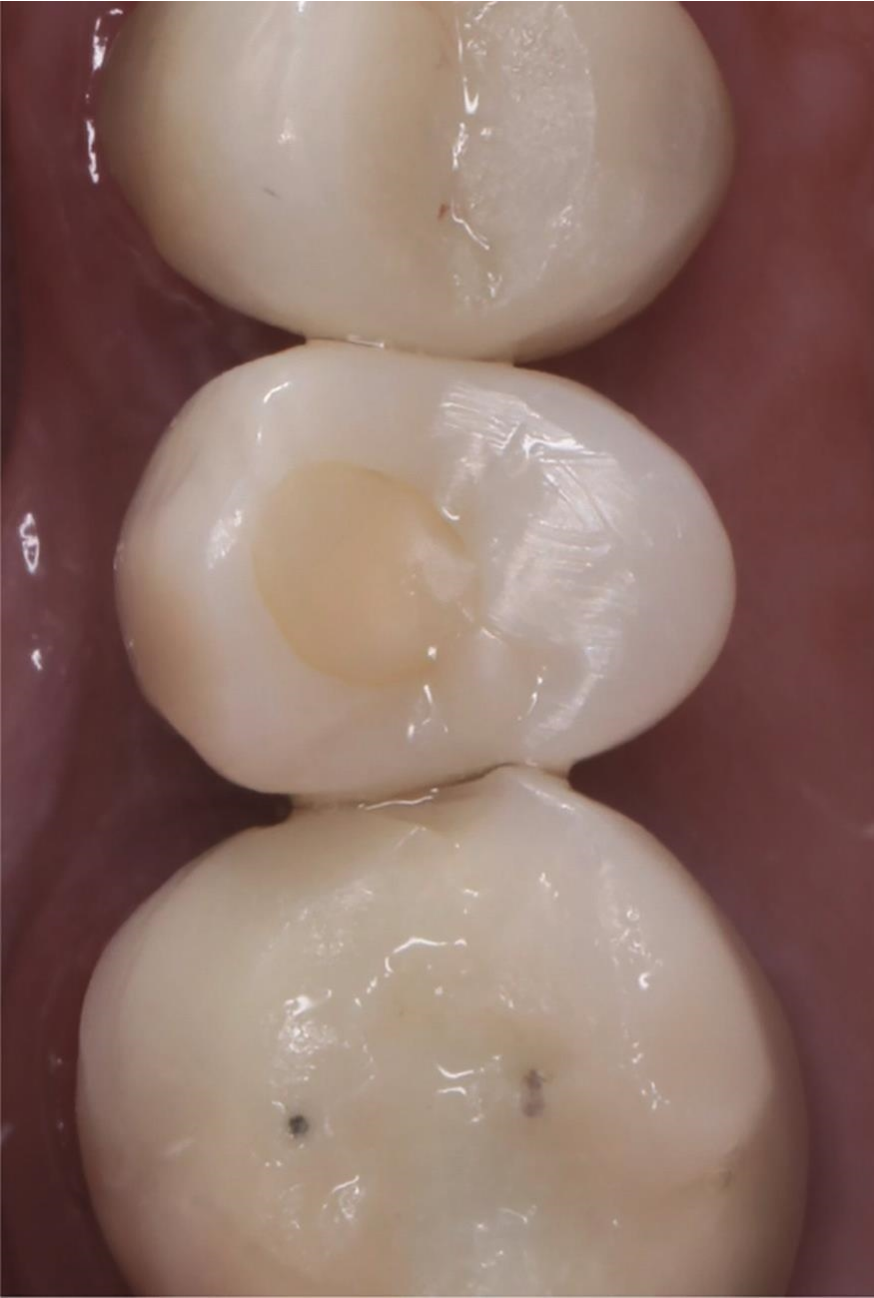
A clinical photograph at the 1‐year follow‐up demonstrating healthy peri‐implant soft tissue, stable crown fit, and satisfactory prosthesis.

**Figure 7:**
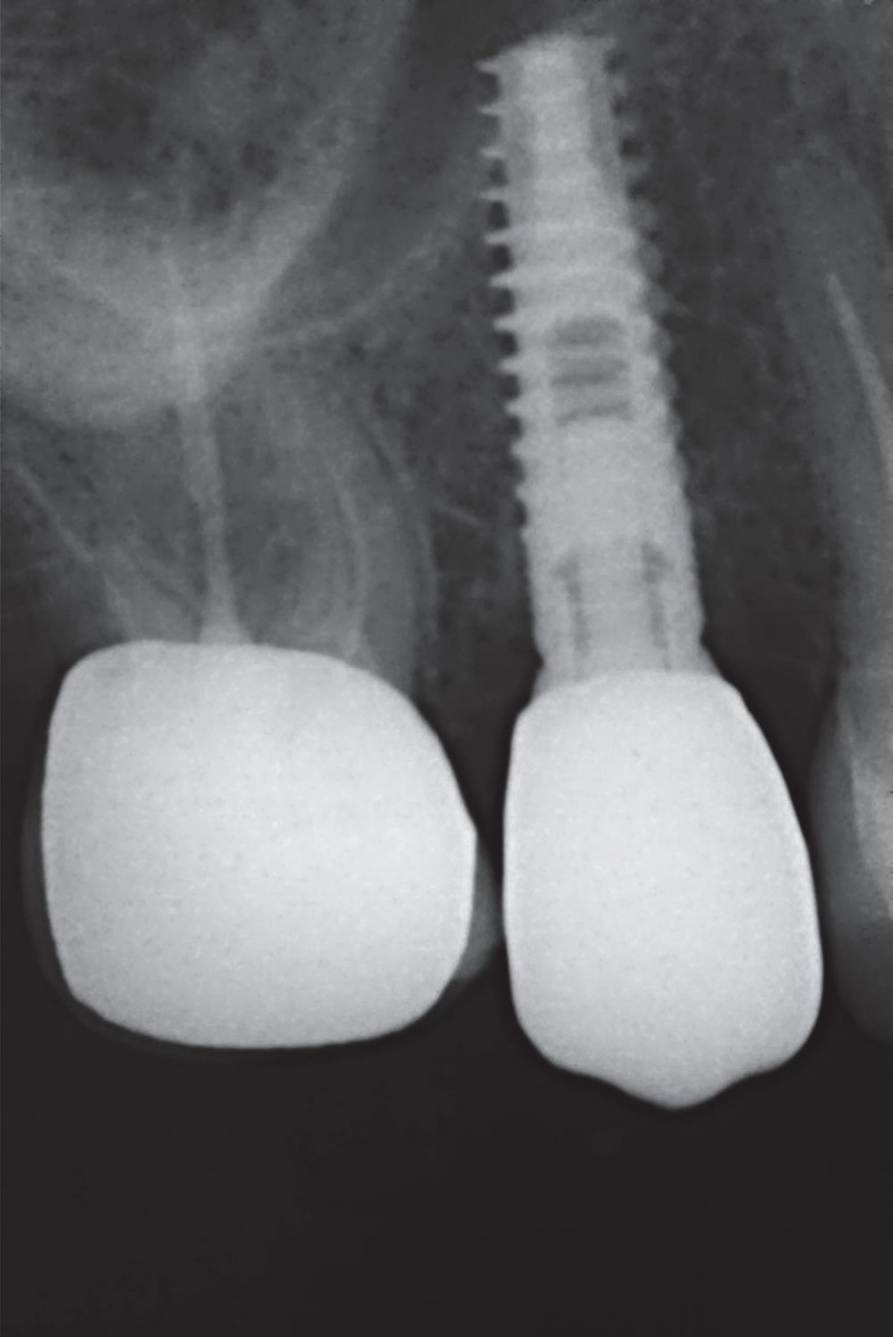
Postoperative radiographic image at the 1‐year follow‐up confirming maintained crestal bone level and accurate prosthesis fit.

At the 1‐year follow‐up visit, the screw‐retained implant‐supported crown remained clinically stable, with no mechanical or biological complications. Clinical and radiographic evaluations demonstrated accurate prosthesis fit, occlusion, and healthy peri‐implant soft tissue (Figures 6 and 7). These findings support the long‐term clinical reliability of the customized impression technique for managing implants with unknown systems.

## 5. Discussion

Cement‐retained implant‐supported crowns may lose retention for several mechanical reasons [9, 10]. Insufficient axial wall height of the abutment is a common contributing factor and often necessitates replacement of both the abutment and the crown. In the present case, however, the existing abutment exhibited adequate axial wall height for retention and resistance after crown removal. Another frequent cause of prosthetic failure is debonding of the crown from the abutment, which has been reported as one of the most common technical complications in implant‐supported single crowns [9]. Additionally, loosening of the abutment screw represents the most prevalent technical complication in single implant restorations [9]. In this case, screw loosening was confirmed after sectioning the cement‐retained crown, supporting the decision to convert to a screw‐retained one.

Removal of a cement‐retained crown presents a clinical challenge. Digitally guided approaches using CBCT and 3D‐printed guides have been described to facilitate precise access to the screw channel [7, 11]. While this technique is effective for patients with a single crown, these methods may be compromised in patients with extensive prosthetic reconstructions due to scattering artifacts from multiple restorations, which reduce CBCT image quality and impair guide accuracy [12]. In the present case, crown sectioning was selected as a simpler and faster approach [13, 14]. Although this technique carries a risk of abutment damage, careful execution minimized this risk and preserved the integrity of the existing abutment.

Closed‐tray implant impressions are often preferred for their simplicity and reduced risk of dislodging the impression post [10]. In addition, there is no screw with long shaft to extrude out of the tray. Therefore, the closed‐tray method was more comfortable to be used in this case. A closed‐tray impression technique, combined with a customized impression post and laboratory analog, allows the precise transfer of the implant’s position and orientation to the laboratory [11].

### 5.1. Advantages of the Customized Technique

Accurate transfer of implant position is crucial for successful dental implant restorations [2, 15]. Standard impression posts and laboratory analogs are typically designed for specific dental implant systems. When the dental implant system is unknown, it can be challenging. In the present case, a customized closed‐tray impression post and laboratory analog allowed precise transfer of the implant’s position and orientation to the laboratory. The three vertical grooves and inclined bevel on the impression post improved rotational stability and facilitated repositioning, while the two horizontal grooves on the laboratory analog maintained its orientation in the master cast.

Several digital techniques, including intraoral scanning and reverse engineering techniques, have been proposed to solve this issue [5, 8, 16–18]. However, these techniques require access to advanced digital equipment and software, which may not be available in all clinical settings. In contrast, the customized technique described is simple, reproducible, and cost‐effective, making it a viable alternative in resource‐limited environments.

### 5.2. Technical Limitations

A key limitation of this method is the absence of internal screw threads in the customized laboratory analog. As a result, the abutment cannot be mechanically secured to the analog and must rely on precise surface adaptation for stability. Any discrepancy at this interface may lead to positional inaccuracies, potentially affecting occlusion or prosthesis fit. This challenge was in consideration during the fabrication of the definitive prosthesis by adding more occlusal contact and then adjusting it clinically. Additionally, the technique requires that the existing abutment and central screw be in good condition, which may restrict its applicability. Careful fabrication and clinical verification are essential to mitigate these risks.

### 5.3. Clinical Relevance and Outcomes

This technique enabled accurate impression‐taking without replacement of the existing abutment. The screw‐retained zirconia crown demonstrated stable function at 1‐year follow‐up, with healthy peri‐implant tissues, proper occlusion, and patient satisfaction. The method is particularly relevant for managing unknown, older, or discontinued implant systems when digital and conventional components are unavailable.

### 5.4. Implications for Future Practice

While effective in this case, further research is needed to evaluate reproducibility across different implant systems and clinical scenarios. Integration of 3D printing and CAD/CAM technologies could enhance precision and streamline fabrication of customized components. Cost‐effectiveness and accessibility make this approach a practical alternative in resource‐limited settings, though careful attention to abutment condition and analog adaptation remains critical.

## 6. Conclusion

This case report demonstrates that a customized closed‐tray impression post and laboratory analog can be used to restore implants with unknown systems. The feasibility of this technique depends on the presence of an intact abutment and central screw, as well as accurate adaptation of the customized laboratory analog. Within these limitations, this technique provides a practical and cost‐effective solution for restoring implants when standard components are unavailable, expanding treatment possibilities for similar challenging cases.

## Funding

The author has nothing to report.

## Consent

Written informed consent was obtained from the patient, in accordance with all applicable laws and regulations concerning privacy and security of personal information, prior to the clinical procedures and the taking of any photographs.

## Conflicts of Interest

The author declares no conflicts of interest.

## Data Availability

The data that support the findings of this study are available upon request from the corresponding author. The data are not publicly available due to privacy or ethical restrictions.
